# Feasibility of Telecom-Wavelength Photonic Integrated Circuits for Gas Sensors

**DOI:** 10.3390/s18092870

**Published:** 2018-08-31

**Authors:** Andreas Hänsel, Martijn J. R. Heck

**Affiliations:** Department of Engineering, Aarhus University, Finlandsgade 22, 8200 Aarhus, Denmark; mheck@eng.au.dk

**Keywords:** photonic integrated circuit, tunable laser, gas spectroscopy, indium phosphide, ammonia

## Abstract

To be of commercial interest, gas sensors must optimise, among others, sensitivity, selectivity, longevity, cost and measurement speed. Using the example of ammonia, we establish that integrated optical sensors provide means to maintain the benefits of optical detection set-ups at, in principle, a lower cost and smaller footprint than currently available commercial products. Photonic integrated circuits (PICs) can be used in environmental and agricultural monitoring. The small footprint and great cost scaling of PICs allow for sensor networks with multiple devices. We show, that Indium Phosphide based commercial foundries reached the technological maturity to enable ammonia detection levels at less than 100 ppb. The current unavailability of portable, low cost ammonia sensors with such detection levels prevents emission monitoring, for example, in pig farms. The feasibility of these sensors is investigated by applying the common noise figures of the multiproject wafer platforms operating around 1550 nm to a model for an absorption measurement. The analysis is extended to other relevant gas species with absorption features near telecom-wavelengths.

## 1. Introduction

There is a growing need for gas sensors in modern society. Not only are they needed for agricultural process control and climate monitoring, but measuring fine concentrations of gasses has medical applications. The analysis of human breath is a non-invasive method to detect diseases [1]. In particular in the case of ammonia (NH${}_{3}$) detection, different sensor concepts reached commercial maturity. None of those solutions has found wide application as NH${}_{3}$ emission monitoring devices for agricultural applications. Sensors to determine ammonia concentrations in the ∼10 ppm sensitivity regime are commercially available [2,3]. While these sensitivity levels are useful to reduce the impact of ammonia on the life stock, emission monitoring, i.e., monitoring ammonia concentrations after the air cleaner, requires more accurate detection systems.

For these applications sensors need to detect NH${}_{3}$ concentrations in the sub-ppm level [4]. Electrochemical sensors approach these sensitivity levels, but suffer from a lack of selectivity and reproducibility. In addition, the poor scalability and longevity prevent widespread application for emission monitoring. Rack-sized laboratory optical spectrometers reach the required detection properties, but are too expensive and not portable, and hence difficult to use for on-site monitoring [5]. Commercial products exceed tens of thousands of Euros per device. Low cost alternatives to laboratory optical spectrometers could consist of tunable single-mode telecom lasers, interaction cells, and photodetectors. The reduced cost of such a device comes at the expense of small tuning ranges, typically of the order of a few nm when considering temperature tuning, and fractions of nm when considering current tuning. The absorption linewidths of gases at room temperature might span more than the tuning range that current tuning can achieve. Thermal tuning can achieve a sufficient wavelength span, but uses a slower tuning mechanism. In any case thermal control is required to ensure that an absorption line is targeted, adding to the device cost. In addition, in environments with many spurious absorption signals a single absorption line might not suffice to identify a gas. Widely tunable telecom lasers, such as Sampled Grating Distributed Bragg Reflector (SG-DBR) lasers, can overcome those problems, but are far more expensive.

**Figure 1:**
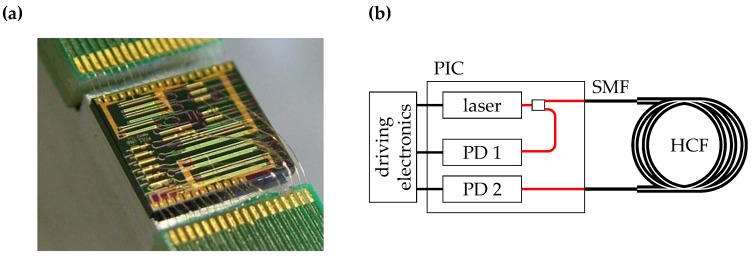
(**a**) Photonic Integrated Circuit (PIC) containing six tunable laser sources. The chip has been attached to a submount and the electrical contacts have been wire-bonded to a printed circuit board. Fibers would approach the light output ports from the left or right side of the chip. These connections can be fixed, e.g., with glue, to provide a packaged device. ©2015 IEEE. Reprinted, with permission, from Latkowski et al.; Novel Widely Tunable Monolithically Integrated Laser Source. *IEEE Photonics Journal* **2015**, *7*, 1–9 [6]; (**b**) Schematic for a compact gas sensor using PICs and a hollow-core fibre (HCF). A HCF can be spliced to lensed or tapered single-mode fibres (SMF), which are typically used for fibre chip coupling on indium phosphide (InP) based, butt coupled PICs. A PIC can contain light sources as well as photodectors (PD 1 and PD 2).

An improvement to such a set-up can be made when considering Photonic Integrated Circuits (PICs), which provide an intermediate solution between the two discussed optical devices (diode based and rack sized equipment). PICs can have a wider tuning range than single mode telecom lasers, and the monolithic integration of laser sources, detectors, and modulators allows for advanced noise reduction methods. PICs could provide a substantial improvement in terms of cost and device size when compared to laboratory equipment, while still being able to span wavelength tuning ranges far in excess of typical absorption linewidths. This work investigates whether PICs achieve the sensitivity levels required for agricultural emission monitoring. The monolithic integration can also increase the stability of the device and make it independent of ambient fluctuations, which is an important feature when operating the sensor outside of laboratories. PICs are scalable for mass production, allowing for sensor networks for distributed gas measurements. Lastly, multiple lasers can be combined on a single PIC, which provides the opportunity for multi-species gas sensors and a very selective readout. Multiple laser sources also open the path to heterodyne detection schemes and provide means to wavelength tracking on chip. Figure 1a shows an example for a PIC with six laser on a $4.7\times 4.1\;{\mathrm{mm}}^{2}$ sized device [6].

Molecular absorption spectroscopy is commonly undertaken at infra-red wavelengths of 2 μm and beyond [7]. Solid state lasers and quantum cascade lasers are able to reach mid-infrared wavelengths, but PICs typically do not reach those wavelengths. Extending the wavelength range PICs can access is still on-going research [8,9]. While a tunable, indium phosphide (InP) based laser operating at 2 μm wavelength has been demonstrated, the tuning range, emission power, and stability does not reach the level of the corresponding circuits at shorter wavelengths [10]. For those shorter wavelengths, the overtones of the optical transitions in the gas need to be measured, leading to much lower absorption coefficients. While spectroscopy using photonic technology at 1.55 μm seems to be unattractive from that perspective, we will show that the performance this technology can compensate for the caveats. Demonstrations of spectroscopy at the ppm level in this wavelength region have already been shown in 2001, but no commercial product has been developed to exploit this research [11]. We will show theoretically by simulations that PICs can reach the same detection levels. While measuring higher concentrations of ammonia might require a redesign of the sensor when using chemical detection methods, e.g., due to saturation effects, optical detection can detect concentrations spanning multiple orders of magnitude [12]. This makes PICs flexible regarding the target market.

The fabrication of PICs requires a clean room, which is a prohibiting factor for many companies, as it is associated to a high entry cost for the technology. While those devices scale very well, i.e., the price per unit reduces when increasing the number of units, initial expenses prevent the development of PICs. This initial barrier can be overcome when facilitating generic technologies and multi project wafer (MPW) foundries [13]. In this framework optical designers can submit a mask layout with predetermined building blocks (BBs) to achieve the desired functionality. The MPW foundries manufacture the chip, allowing PICs to be used without operating a clean room. The chips can be packaged to allow for turnkey operation, further reducing the demands on the measurement set-up. Several foundry, design and packaging services are brokered by JePPIX [14].

This work is dedicated to the study of feasibility of PICs for ammonia sensing, but can be extended to other gas species in the PIC wavelength range. This includes N${}_{2}$O, CO${}_{2}$, CO, H${}_{2}$S, C${}_{2}$H${}_{2}$, and CH${}_{4}$. Several of those gasses are climate relevant and could be included in emission tests. Additionally, CO${}_{2}$ is monitored to determine ventilation requirements in animal farms to ensure the health of the life stock. The strongest absorption lines of those gases in the accessible wavelength range are outlined in Figure 3. As the Figure shows, e.g., CH${}_{4}$ concentrations of 100 ppm can be detected in the same fashion.

## 2. Absorption Strength and Signal Levels

The requirements on the detection system depend on the signal strengths of the gas sample. For a direct absorption measurement, the signal corresponds to the amount of light that is absorbed when passing through the medium. We used the values obtained from the HITRAN database to link concentrations to absorption line strength [15,16]. We specifically targeted absorption lines in the C-band of optical telecommuncations (1.53–1.565 μm), as PICs have reached a high maturity in this region. Several other absorption lines can be found in the long-wavelength end of the S-band (1.46–1.53 μm). The strongest absorption signals were found at the wavelengths of 1.512, 1.514, 1.522, 1.527, and 1.531 μm. All of the investigated lines suffer from overlapping absorption lines with other gases present in the atmosphere, with water vapour providing the strongest spurious signals. The presented absorption values are for gas mixtures with a pressure of 1 atm, a temperature of 296 K and a propagation length of 1 m. The exact gas composition is listed as *USA model, mean latitude, summer, H = 0* in [16]. These values only provide approximations for the gas composition, which can differ depending on where and when measurements are taken. Several constituents of the atmosphere have changed their respective concentration, such as in the case CO${}_{2}$, whose increased concentration over the last years and is linked to climate change [17].

**Figure 2:**
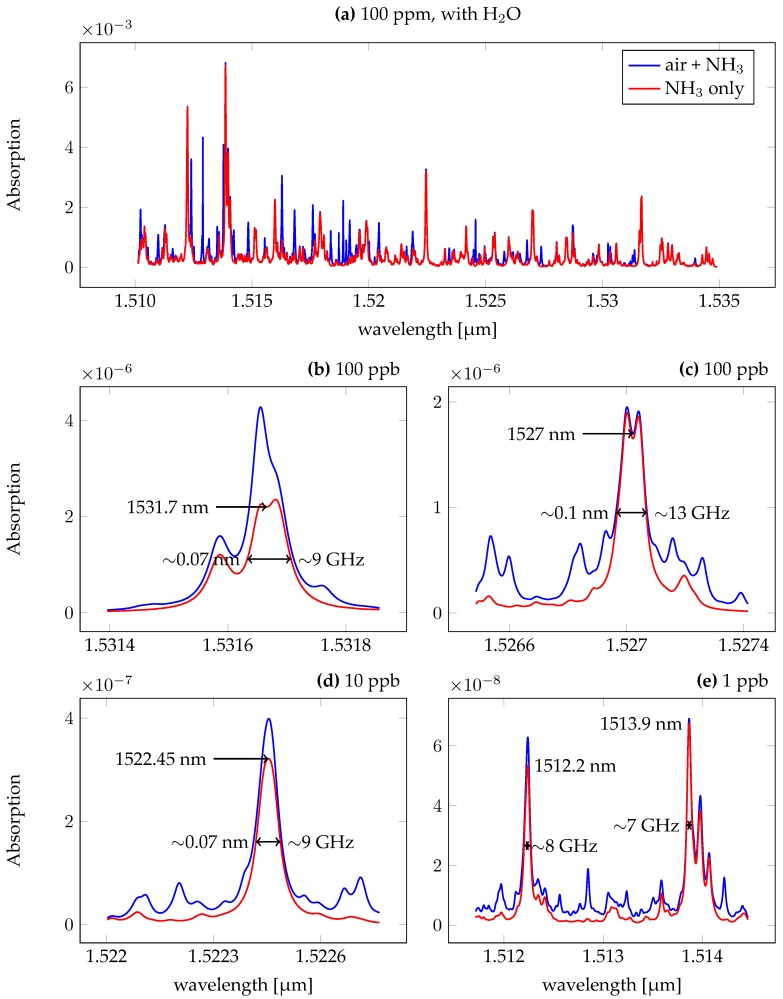
Absorption spectra for ambient air mixed with NH${}_{3}$. (**a**) Overview of the available absorption lines. The red line shows the contribution of the 100 ppm added NH${}_{3}$ to the total absorption spectrum, which itself is shown in blue. The majority of the interfering signal is caused by water vapour. (**b**–**e**) After the removal of water, different absorption lines can be exploited for spectroscopic purposes. While the strength of all the lines is comparable, the overlap with other lines as well as the distance to the design wavelength of the on-chip laser can make certain lines more favourable compared to others. The sensitivity limits due to line overlap are shown in Figure 3.

**Figure 3:**
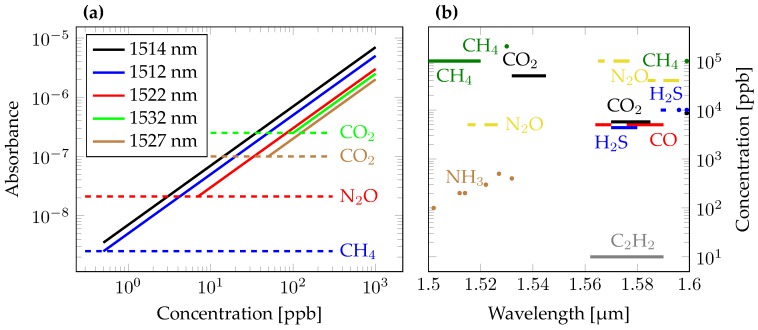
(**a**) Absorbance values for different concentrations of NH${}_{3}$ in atmosphere for the investigated absorption lines and a propagation length of 1 m. The lower concentration limit is given by the absorption profiles of other gases in the atmosphere that overlap with the NH${}_{3}$ lines. The limiting gases have been linked to the corresponding ammonia absorption lines with a dashed line. For this plot water vapour has been removed from the ambient air. (**b**) Detectable concentrations and wavelength ranges of different climate relevant gas species, assuming absorption signals of ∼$10^{-6}$. Lines in the diagram represent a close set of multiple usable lines, whereas dots refer to single absorption lines.

For a proper gas detection we require the contribution of NH${}_{3}$ to be at least 50% of the total absorbance at the peak wavelength. Concentrations of 10 ppm can be detected without the removal of water vapour at 1.531 μm. The absorption strength can be linked to the NH${}_{3}$ concentration by

(1) $$A∼10^{-8}\;m^{-1}{\mathrm{ppb}}^{-1},$$

for all the mentioned absorption lines. The exact absorption value for the individual lines differs by not more than a factor of 5, which allows for this approximation. At lower concentrations of NH${}_{3}$, water vapour will provide the dominant absorption signal. If water can be removed, e.g., by cooling the gas below the condensation point of water, lower concentrations can be measured, before other gases, e.g., CO${}_{2}$, dominate the absorption signal. The impact of water vapour absorption and hence the importance of removing water from the gas mixture is enhanced for the shorter wavelengths that we investigated. It is worth mentioning that water vapour also affects the performance of other available highly sensitive gas sensors and is not a problem unique to the optical detection. Figure 2 shows the signal strengths for the different absorption lines. Five lines in the spectrum have been selected due to their strong absorption in comparison the other lines. The figure also shows the corresponding lineshapes and the absorption background caused by other gases in the atmosphere. Figure 3a shows the resulting concentrations for each line that can be reached before those spurious signals impede the analysis. All of the lines can in principle be used to measure concentrations higher than 100 ppb, with a few lines allowing for concentrations as low as 1 ppb. A similar analysis can be done for other gases, which feature absorption lines in the investigated wavelength range. An estimate for the concentrations corresponding to a relative absorption of $10^{-6}$ for several such gases is shown in Figure 3b. The limits in Figure 3b are not given by spurious signals, unlike in Figure 3a, but by the absorption strength. Figure 3 assumed 1 m light propagation in the medium, and total overlap of the light field with the analyte gas. Such conditions can be reached with HCFs. On-chip gas cells for evanescent sensing in InP can be estimated to confine between 1% and 10% of the light power in the gas interaction region, and reach propagation lengths of ∼10 cm, resulting in 100 to 1000 times weaker signals [18,19]. The detectable gas concentrations increase correspondingly by the same factor. Complex structures, such as pillar based photonic crystal waveguides, can potentially improve the in-gas confinement factor, but are not manufactured in a standardised process for mass production [20].

The analysis above assumes a single mode laser, i.e., light is emitted at a singular wavelength. Real laser systems have a finite bandwidth, as well as sidebands in their emission profile. Single-mode lasers are typically evaluated regarding their linewidth in full width at half maximum (FHWM), the side mode suppression ratio (SMSR), and their tuning range. With typical linewidths around 1 MHz, semiconductor lasers are suitable, as the absorption lines have linewidths of multiple GHz. The finite linewidth of the laser is consequently expected to be of negligible impact. Empirically, it has been found that an SMSR of 30 dB is required for spectroscopic measurements [21]. It can be reasoned, that a similar requirement needs to be reached here. In dry air, the strongest spurious absorption signals can be found at wavelengths of 1530 and 1570 nm, with signal strengths of ∼7 × 10^−6^ and ∼6 × 10^−5^ respectively. The relevant ammonia absorption lines are at wavelengths of 1531 nm, or shorter. As such it can be stated, that for an absorption line with a strength of ∼$10^{-7}$ (corresponding to 10 ppb of NH${}_{3}$), the close neighbour SMSR has to be greater than 20 dB, and for lines further away greater than 30 dB. This, of course, only holds when considering a single side mode polluting the signal, at the worst possible wavelength. Performance levels exceeding those requirements have been reported in numerous publications [6,10,21,22]. The requirements are further relaxed when dealing with higher concentrations and stronger signals. This is, in many aspects, a worst case estimation, as the strongest laser side mode does not necessarily overlap with the strongest spurious absorption line. Similarly the typical tuning ranges do not pose a limit, as in theory a measurement over a single absorption line suffices. In the same platform that is used here, tuning ranges of more than 70 nm have been reported [6]. The same publication also showed that fine tuning over an absorption line is possible. A wider tuning range allows for spectroscopy over multiple absorption lines. Such a detector can show improved selectivity, as lines overlapping with other gases in the mixture can be ignored, and enables multi-species gas spectroscopy [23].

## 3. Optical Detection Methods

A minimal detection system for absorption spectroscopy consists of a light source, an interaction cell, and a photodetector. When choosing a broadband light source, the detection system needs to contain a spectrometer to extract spectral data. Alternatively a tunable single mode laser can be paired with a regular, broadband photodetector. Sweeping the laser wavelength over an absorption line yields the spectral information. As the output power can fluctuate when tuning the wavelength, a second photodetector is typically used to normalise the transmission with respect to the laser output power. A two detector system scheme also allows for balanced detection, which only measures the signal differences between two detectors.

For PICs in InP, tuning a laser and using a broadband detector is the more attractive option. The requirements on the wavelength selectivity of the filter are reduced, as the filter is effectively passed through multiple times when placed inside the laser cavity. In addition, there has already been substantial work in laser design, making the design process very well understood. Silicon (Si) photonics features potentially lower waveguide losses and a more mature clean room processing, but lacks the active elements, such as optical amplifiers [19]. On-chip spectrometers have been demonstrated, which can be used for spectroscopic purposes [24,25]. An advantage of using this approach would be the accessibility of different wavelength regions with possibly stronger absorption lines. With hybrid or heterogeneous integration, Si circuits can have access to compact optical amplifiers on the same chip [26].

With an increasing number of devices, the advantages of PICs in comparison to systems consisting of discrete components are more and more visible, as most of the functionalities can be implemented in the same monolithically integrated circuit.

In contrast to detector and light source, the interaction cell cannot easily be integrated on the same chip. The interaction cell contains the volume of the gas sample that can interact with light, here to measure the absorption profile. Low concentrations, whose detection is the aim of the spectrometer, result in few absorbing molecules in a given volume. In set-ups with discrete components resonant cells and multi-pass cells found applications to increase the optical path length in the gas and hence the interaction. The alignment of resonant and multi-pass cells is more complex than for single path cells and can lead to beam steering instabilities and added noise. The low quantity of absorbers requires long optical path lengths through the gas sample, which is typically difficult to achieve on-chip. Measurements for concentrations in the sub-100 ppm regime in Methane have been demonstrated recently, using a silicon photonic chip, operating at 1650 nm [27]. Evanescent sensing with on-chip gas cells requires low-loss waveguides and large confinement of light in the cladding area, i.e., outside of the waveguide [26]. In InP, propagation lengths are limited to a few centimetres, due to the waveguide losses (∼1 dB/cm) [19]. Si PICs can feature propagation losses of less than 1dB/m, but still suffer from a low confinement of light in the gas. Lastly, chip sizes in the mm regime are geometrically unable to reach waveguide lengths of multiple meters. When relying on resonant structures, the effective interaction lengths can be stretched, at the expense of the stability regarding ambient fluctuations. Both effects, lower light confinement in the gas volume and short propagation lengths, impede the absorption signal strength and hence the detection levels. On-chip interaction cells in InP have to our knowledge only been used for fluid sensing, and not for highly sensitive gas spectroscopy [18].

In InP PICs, light is typically emitted from the facet of the laser chip can be coupled to a fibre, which allows for using fibre-based gas cells instead of on-chip gas cells. Fibre-coupled gas flow cells with propagation lengths of up to 80 cm are commercially available, with fibre-to-fibre throughputs of roughly 35% [28]. In those cells light is coupled into free space in the gas cell and coupled back into the fiver at the other end of the cell. Hollow-core fibres (HCFs) and hollow-core waveguides are already commercially available [29]. Off-the-shelf HCFs can confine ∼98% of the light in the target gas while maintaining an attenuation of less than 20dB/km at 1550 nm, and as such provide similar performance as propagation in free space at a much reduced footprint. Filling and light coupling complicate the creation of HCF based flow cells. Filling the HCF requires openings for the gas to enter the fibre, which are typically found at the end of the fibres. When splicing the HCF to a single-mode fibre (SMF), an open ended fibre is not an option any longer. Holes can be placed at the fibre splice, or placed along the HCF. Both methods are part of on-going research and reduce the overall performance of the fibre, e.g., due to back reflections or leaky modes [30,31]. Light coupling on the other hand is complicated due to possibly mode mismatch between HCF and SMF. Even if both fibres are single moded, mode field mismatch can cause reflections or coupling losses. Nevertheless, NH${}_{3}$ spectroscopy in HCFs has already been demonstrated, although not for trace gas detection [32]. In this case light has not directly been coupled from fibre to fibre, but used free space optical components in between. When directly a single mode fibre to a hollow core fibre, no free space element is required, which is expected to increase the stability of the setup, assuming aforementioned caveats can be mitigated. A schematic for such a setup is shown in Figure 1b.

Hodgkinson and Tatam provided an extensive review for optical gas sensing methods [12]. Out of the many techniques discussed in their review, we focus on the tunable laser absorption spectroscopy, and the different methods to obtain the signal within this scheme. Other promising techniques, e.g., photo-acoustic spectroscopy, would increase the complexity of the circuit and will not be discussed here. This does not mean that those techniques cannot benefit from photonic integration. Acoustic sensors have already been demonstrated on chip, which could be co-integrated with a tunable laser source [33].

The first method is the direct absorption spectroscopy (DAS). Light that is sent through the interaction cell experiences absorption, which will be extracted by monitoring the change in transmission directly with a photodetector. The signal is kept at one wavelength while the detector takes the value, before the wavelength is changed. Conceptually, this is a simple and straight-forward detection scheme, and consequentially comparatively simple to optimise. When scanning over an absorption line, this method is also calibration-free, i.e., no calibration with a known sample is required.

The laser light can also be modulated during the measurement, resulting in a more complex data treatment, but with the potential to eliminate some noise signals. A sinusoidal wavelength modulation centered at the absorption peak can be used for wavelength modulation spectroscopy (WMS), the second method discussed here. The modulated signal is read out with a lock-in amplifier with a matching frequency. Alternatively higher harmonics can be looked at leading to a weaker signal, but to a better background removal. The added complexity of the setup is rewarded with suppression of low frequency noise, such as 1/f noise. Low frequency noise prevents improving the signal to noise ratio when averaging and is a bottleneck for measurements at lower bandwidths. WMS and Frequency Modulation Spectroscopy (FMS) are closely related and differ in regards to their modulation strength, i.e., the wavelength range that is tuned, and the modulation bandwidth, i.e., how fast a tuning cycle over the wavelength span is done [34]. FMS typically describes a very fast modulation with low modulation strength, which can be done on-chip with phase modulators. WMS describes a stronger, but slower modulation, by scanning the emission of the laser over the absorption line. This can be done using phase modulators within the laser cavity, or by increasing the current of the laser diode driver, which also has an impact on the emission wavelength. The strength of the noise suppression depends on the details of the experimental setup and is discussed by Silver [35]. As the required modulation bandwidths scale with measured absorption linewidth, FMS would require a modulation of several tens of GHz (see Figure 3). As this can be challenging for the measurement electronics, only WMS has been considered here. However, it is worth mentioning that FMS can also obtain the dispersion of the gas sample, which could provide a stronger effect than the absorption in some cases. The details of the noise suppression are system dependent, we simplified the evaluation by assuming the best case condition, i.e., full suppression of the 1/f noise of the laser source.

**Figure 4:**
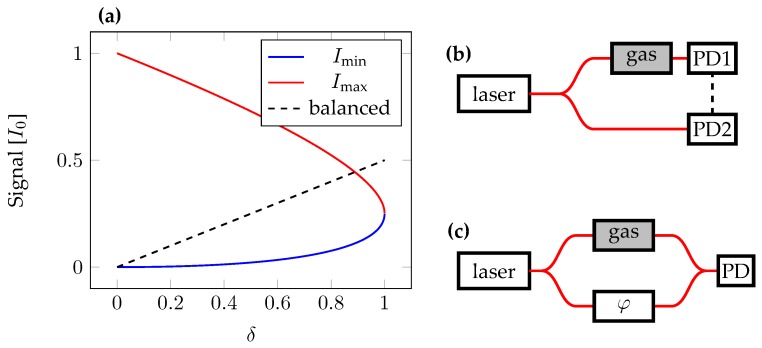
(**a**) The minimum intensity signal, shown in blue, from the homodyne detection is always lower than the signal that could be obtained from the balanced detection scheme, shown dashed and in black. In that regard direct homodyne measurements do not improve the sensitivity of the gas detection. The maximum intensity is plotted in red. (**b**) Schematic for a balanced detection system. The difference signal between both detectors (PD1 and PD2) is read out. (**c**) Schematic for a homodyne detection system. The interference of both arms is measured to obtain the absorption. With the phase modulator (φ) the phase can be tuned to obtain the minimum power on the photodetector (PD).

As a third method a balanced detection scheme can be applied. In this case the difference signal between the reference and measurement photodetector is read out. A balanced detection setup with distributed devices can detect ammonia concentrations as low as 0.7 ppm, as has been shown by Claps et al. in 2001 [11]. Those measurements were taken at a low pressure, 100 Torr, with an optical path length of 36 m. The seven-fold increase of pressure when measuring gasses in ambient air results in line broadening, which distributes the optical power over a wider wavelength range, but leads to a higher absorber density and hence to a stronger absorption. Balanced detection potentially eliminates laser noise, but distributes light over two detectors reducing the signal strength. A tunable attenuator or modulator is required to ensure optimal performance, as signal and reference detector need to receive the same optical power for optimum performance. A time delay between both detector parts in the balanced detection provides a bandwidth limit for the suppressed noise. In PICs, detectors, modulators, and attenuators can be integrated on the same chip, which allows for a compact detector system and reduces the distance between components, e.g., detectors for balanced detection. Balanced detection is especially attractive, as the reference detector does not need to couple light out of the chip, which is a major cause for losses in the measurement. Coupling losses from fibre to chip and vice versa can reach 3 dB, or 50%. In balanced detection two detectors are used to read out the signals of both arms. Interfering both signals on the same detector, results in homodyne detection. Changing the optical phase in one arm tunes the interference. When tuning over a full period, maximum and minimum intensities of the interfering light can be obtained ($I_{\max}$ and $I_{\min}$). The minimum intensity $I_{\min}$ rises with increasing absorption according to $I_{\min}\approx \delta^{2}\frac{I_{0}}{16}$, similar to balanced detection with $I_{\mathrm{balanced}}=\delta \frac{I_{0}}{2}$. Here, a weak relative absorption δ<<1 has been assumed, where $I_{0}$ denotes the laser power, which is in both cases equally split into signal and reference ($I_{1}$ and $I_{2}$). When comparing the signal strength of balanced detection and tracking the minimum interference power, balanced detection has a stronger effect, which can also be seen in Figure 4. The figure has been made without approximation regarding the absorption strength, which takes the minimum and maximum values of the equation $I=\left(I_{1}+I_{2}+2\cos\Delta \phi \right)/2$, where Δϕ is the phase difference between both arms. Since homodyne detection provides a weaker signal while simultaneously increasing the complexity of the PIC as a phase tuning element is required, it has not been further investigated in this work. Nevertheless, PICs do allow for on-chip phase tuning elements and interferometric detectors, and can be used in this configuration if wanted.

## 4. Noise in the Detection Systems

Noise can be defined as “unwanted disturbances superposed upon a useful signal that tend to obscure its information content” [36]. While a weak signal can be amplified to be detectable, interfering noise will be amplified as well. A signal needs to be reasonably strong in relation to its noise to allow for accurate measurements. We consider a signal-to-noise ration (SNR) of 1 to define our detection limit. That means that the absorption strength has to be at least as strong as the noise strength, which determines the NH${}_{3}$ concentrations that can be measured.

Noise can be classified according to its source, which would entail the laser source, the interaction cell, and the photodetector. As the interaction cell cannot be part of the PIC, it is not included in our calculations. If the interaction cell is the limiting component, then it is so for all optical spectroscopes; integrated or not. Laser source and photodetector on the other hand, can and should be part of the PIC. We used the parameters provided by the MPW foundry SMART Photonics to assess the noise performance for the different methods [37].

We limit our measurement bandwidth δf to frequencies much lower than the typical relaxation oscillations in InP lasers. In this case, it is reasonable to consider shot noise and electronic noise from the laser diode driver as the remaining laser noise sources. The laser shot noise current as seen by the photodetector can be calculated with

(2) $$i_{\mathrm{laser},\mathrm{shot}}=\alpha \sqrt{2h\nu P_{\mathrm{laser}}\delta f}.$$

where hν corresponds to the energy per photon, and $P_{\mathrm{laser}}$ to the output power of the laser. This noise is attributed to the quantisation of the light, which emits energy quanta corresponding to the energy of a single photon. In accordance with the values specified by SMART Photonics we assumed a laser output power of 20 mW. This is not at the top end of possible output powers, as commercial products can reach output powers of ∼100 mW [38]. Dedicated circuits should be able to reach competitive levels, even when relying on MPW designs. The factor α in Equation (2) contains the responsivity of the photodiode (0.85 A/W), which links the light emission to a measured photocurrent, as well as losses due to fibre-chip coupling and propagation in the fibre. Noise from the current source that drives the optical amplifier section has a large contribution to the laser noise. This is technical noise and linked to the quality of the used current source. For our calculations we used the noise figures from the Koheron DRV200 (Orsay, France). With a cost of ≤275 $ this is in line with the goal of cheap and compact sensor [39]. Laser diode driver noise is frequency dependent, in contrast to the white noise emitted from the other investigated noise sources. Lower frequencies contribute stronger to the total noise current than higher frequencies, which is the reason why WMS can be used to reduce its impact. Multiple vendors offer ultra-low noise laser diode drivers, which will reduce the impact of the current source to the overall noise. Unfortunately most vendors do not provide detailed data about the frequency dependence of the noise current, but only the integrated noise from DC to 10 kHz; e.g., less than 70 nA for the LDX-3620B (Irvine, USA) from Newport [40].

The detector suffers from a noise associated to the quantisation of charges. The corresponding detector shot noise is described by

(3) $$i_{\mathrm{pd},\mathrm{shot}}=\sqrt{2eI_{pd}\delta f},$$

with $I_{pd}$ as the current measured by the photodetector, here 3.4 mA, and *e* as the electron charge. Since the noise increases with the square root of the photo current, while the signal strength increases linearly, a higher laser output power would result in a better signal-to-noise ratio. For very high laser intensities the photodetector saturates, and close to saturation nonlinear behaviour can be expected. Here this is not assumed to be limiting, as the saturation point can be increased by design, such as increasing the cross section of the photodiode. Photodetectors typically exhibit a dark current, which is also subject to quantisation noise. With a dark current of $I_{\mathrm{dark}}$ of 20 nA it can be calculated with

(4) $$i_{\mathrm{dark}}=\sqrt{2eI_{\mathrm{dark}}\delta f}.$$

While increasing the cross section of the photodiode is expected to extend the linear operation regime of the detector, it is accompanied by a higher dark current. In the configuration investigated in this study, dark noise can be neglected, $I_{\mathrm{dark}}<<I_{pd}$.

The Brownian motion of the electrons in a conducting material causes thermal noise. This noise strongly depends on the temperature *T* and the load resistance *R*, as can be seen from

(5) $$i_{\mathrm{thermal}}=\sqrt{4\frac{k_{B}T}{R}\delta f}.$$

Here, $k_{B}$ is the Boltzmann constant. The thermal noise was calculated for a load resistance of 50 kΩ. The load resistance has an impact on the bandwidth of the detector, and for a very fast acquisition speeds exceeding 10 kHz, a lower load resistance can be required. The used parameters have been summarised in Table 1.

**Figure 5:**
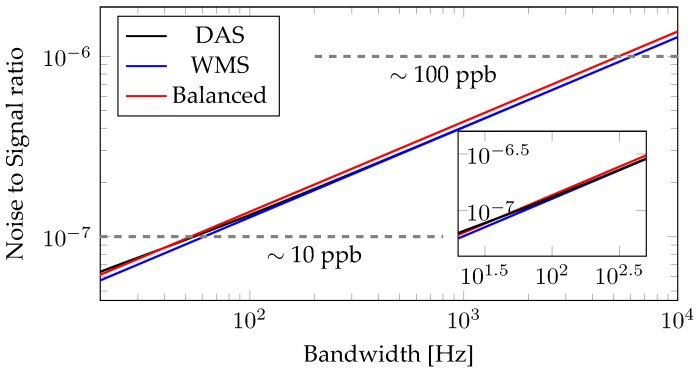
Relative noise levels for different bandwidths for the three investigated methods. While direct absorption spectroscopy (DAS, shown in black) has the hightest noise contributions at lower bandwidths due to the 1/f noise of the laser diode driver, it approaches the level of WMS (blue) for higher bandwidths, which compensates for this noise contribution. The balanced detection scheme (red) compensates for all the laser noise, and shows a similar trendline as wavelength modulation spectroscopy (WMS). The inset shows a zoom in of the plot and highlights, that at bandwidths of 100 Hz all the methods show a similar performance. Line markers are added to indicate the expected sensitivity levels for a SNR of unity and a propagation length of 1 m.

We calculated the expected noise values for three different methods: DAS, balanced detection, and WMS. In DAS, all noise sources contribute with their full strength. Similarly the full output power can be used to gain the absorption signal. In balanced detection, only one half of the light contains an absorption signal, but noise originating from the laser is removed. WMS suppresses the noise contributions that fall off with higher bandwidth, such as 1/f-noise. The outcome of all three calculations and the respective sensitivity levels are shown in Figure 5.

## 5. Results

Assuming a signal strength matching the overall noise of the system, Figure 5 and Equation (1) allow to determine which concentrations of ammonia can be detected.As can be seen, all the investigated methods are capable of detection levels below 100 ppb for bandwidths of 1 kHz, that means one thousand data points per second. All the methods show a similar performance, especially at higher bandwidths where the disadvantage of DAS due to the 1/f-noise is mitigated. For bandwidths of less than 100 Hz balanced detection and WMS show a superior performance. While WMS needs a lock-in amplifier, balanced detection can be implemented with almost no additional costs. This allows for ammonia sensor with sensitivities of 10 ppb or better, surpassing the requirements for agricultural applications. Very short acquisition times might be pointless, as the fill time of the gas cell can be multiple seconds long, if not minutes. The similar performance of all methods is an indicator, that there are multiple noise contributions of comparable strength. While DAS is limited by the noise originating from the laser diode driver, shot noise on the photodetector is the limiting factor for the balanced detection scheme. The former can be reduced when employing a low noise laser diode driver, the latter by improving the output power of the laser system, or by minimising losses in the gas-filled fibre. This results confirm the thermal noise as main noise source for low laser power, and shot noise at higher power levels, assuming that the laser driver noise has been eliminated [35]. Noise contributions from the gas cell, such as coupling or beam steering instabilities, have not been investigated.

Depending on the selected wavelength window, concentrations down to the ppb level can be measured before spurious signals from other air constituents pollute the signal. To reach those limits, water needs to be removed from the ambient air. This is fairly standard for ammonia detection in air, and optical sensors are not the only systems suffering from it. Water vapour can effect the performance of chemical sensors. Optical sensors, on the other hand, can still take meaningful measurements, if a proper wavelength calibration is undertaken. Most of the investigated absorption lines have water lines very close to the absorption line, but not directly on top of them. While water vapour might provide the strongest signal when scanning over wide wavelength ranges, if limiting the system to only the targeted absorption line, data can still be obtained. Life wavelength calibrations can be done when including wavelength reference cells in the overall detection system. This option has not been explored in this work.

## 6. Discussion and Conclusions

The current need for ammonia sensors for emission monitoring in agricultural cannot be answered with currently commercially available devices, as they are either not sensitive enough, or too expensive to allow the deployment of sensor networks. With the possibility of detecting NH${}_{3}$ at concentrations of less than 100 ppb in ambient air, PICs can be the solution. Not only do they scale with higher device numbers enabling whole sensor arrays, but also is the underlying optical absorption spectroscopy adaptable to different gas species and sensitivities. All of the investigated detection methods reach the sensitivity needed for emission monitoring. Numerical methods for noise reduction can further improve the sensitivity [41].

PICs allow for complex optical circuits on a single chip. Balanced detection and the application of modulation techniques have a negligible impact on the costs or footprint of the PIC. The true increase in complexity originates from the electrical processing of the data, as each detector needs an electrical readout, while balanced detection or WMS require complex electrical circuitry. This bottleneck has already been identified by the scientific community, and current research regarding flip chip technology, which allows for integration of complex electrical and optical circuits, is expected to remove this obstacle in the near future. Independent of this development, PICs can already provide an access to affordable gas sensors for emission monitoring, such as highlighted here on the example of ammonia detection. While particular focus in the analysis was given to ammonia sensing, a selection of other relevant gases has been presented, that can also benefit from photonic integration. To move the PIC based gas sensor development to industry, the scientific community needs to develop demonstrators, e.g., using MPW technology, and address difficulties with packaging or light coupling to the interaction cell. Current progress on chip based interaction cells paves the way for completely integrated gas sensors in the near future [27].

## Figures and Tables

**Table 1:** Parameter list used in the simulations.

Parameter	Value	Remarks
Laser power	20 mW	on-chip
fibre coupling losses	50%	-
additional fibre losses	20%	e.g., splices, propagation losses
total losses	80%	2 × coupl. losses + add. losses
Responsivity of PD	0.85 A/W	-
Resulting photo current	3.4 mW	-
dark current	20 nA	-
load resistance	50 kΩ	-
Temperature	296 K	-
laser driver noise	-	see [39]
Slope efficiency	∼0.15 W/A	-
